# Characterization of preneoplastic and neoplastic rat mesothelial cell lines: the involvement of TETs, DNMTs, and 5-hydroxymethylcytosine

**DOI:** 10.18632/oncotarget.8970

**Published:** 2016-04-25

**Authors:** David Roulois, Sophie Deshayes, Marie-Noëlle Guilly, Joëlle S. Nader, Charly Liddell, Myriam Robard, Philippe Hulin, Amal Ouacher, Vanessa Le Martelot, Jean-François Fonteneau, Marc Grégoire, Christophe Blanquart, Daniel L. Pouliquen

**Affiliations:** ^1^ CRCNA, Université d'Angers, Université de Nantes, Nantes, France; ^2^ INSERM, Université d'Angers, Université de Nantes, Nantes, France; ^3^ CNRS, Université d'Angers, Université de Nantes, Nantes, France; ^4^ CEA, DSV, iRCM, LCE, BP6, Fontenay aux Roses cedex, France; ^5^ Cellular and Tissular Imaging Core Facility (MicroPICell), Nantes, France

**Keywords:** mesothelioma, preneoplastic mesothelial cells, rat, TETs, DNMTs

## Abstract

Malignant mesothelioma (MM) is one of the worst cancers in terms of clinical outcome, urging the need to establish and characterize new preclinical tools for investigation of the tumorigenic process, improvement of early diagnosis and evaluation of new therapeutic strategies. For these purposes, we characterized a collection of 27 cell lines established from F344 rats, after 136 to 415 days of induction with crocidolite asbestos administered intraperitoneally. Four mesotheliomas were distinguished from 23 preneoplastic mesothelial cell lines (PN) according to their propensity to generate tumors after orthotopic transplantation into syngeneic rats, their growth pattern, and the expression profile of three genes. PN cell lines were further discriminated into groups / subgroups according to morphology in culture and the expression profiles of 14 additional genes. This approach was completed by analysis of positive and negative immunohistochemical MM markers in the four tumors, of karyotype alterations in the most aggressive MM cell line in comparison with a PN epithelioid cell line, and of human normal mesothelial and mesothelioma cells and a tissue array. Our results showed that both the rat and human MM cell lines shared in common a dramatic decrease in the relative expression of *Cdkn2a* and of epigenetic regulators, in comparison with PN and normal human mesothelial cells, respectively. In particular, we identified the involvement of the relative expression of the Ten-Eleven Translocation (*TET*) family of dioxygenases and *Dnmt3a* in relation to the 5-hydroxymethylcytosine level in malignant transformation and the acquisition of metastatic potential.

## INTRODUCTION

Malignant mesothelioma (MM) is a rare, aggressive cancer mainly related to asbestos exposure [1], the long latency time between exposure and occurrence of clinical symptoms limiting the efficacy of therapeutic interventions. Given its chemoresistance, current therapies have a negligible impact on overall survival due to a lack of understanding of the complex biology of MM [2]. Thus, a better understanding of the different steps in the development of this disease should be of great interest for the identification of early markers of MM and of other potential therapeutic targets. In addition, to improve the evaluation of new therapeutic strategies, well-characterized and highly relevant preclinical MM models are urgently required.

Cell lines are universal model systems in cancer research and their characterization has provided crucial insights into the mechanisms leading to malignant transformation. Nevertheless, only a small number of cell lines meet the minimum standards of authentication and characterization [3], emphasizing the need to avoid the use of cultures at high passage levels [4]. Preneoplastic cells represent crucial elements in understanding the timing and sequence of events that lead to neoplastic transformation. Downregulation of E-cadherin was observed in the preneoplastic lesions of a rat model of lung carcinogenesis, which preceded the occurrence of the epithelial-to-mesenchymal transition (EMT) [5], a key feature of the process of evading growth suppressors. Among the new hallmarks identified during the last decade [6], deregulation of cellular energetics, such as local hyperinsulinism, has also been identified to lead to the development of preneoplastic lesions [7], while other reports have demonstrated links between glucose metabolism and epigenetic regulator function [8].

The last decade has also been characterized by a considerable improvement in our understanding of the consequences of epigenomic disruption on the genesis of cancer [9]. Among the multiple epigenetic regulators associated with gene repression, the Ten-Eleven Translocation (TET) family of dioxygenases, which catalyze the conversion of 5-methylcytosine (mC) to 5-hydroxymethylcytosine (hmC), have received much attention. hmC levels were also found to be remarkably depleted in different human cancer tissues relative to their normal counterparts [10], while TET1 was downregulated [11]. Recent reports have suggested that TET2 could be the major function affected in myeloid disorders and that loss of TET2 catalytic function might promote leukemogenesis [12]. To date, all studies investigating hmC levels and/or *TET* expression have systematically compared tumor tissues from various origins relative to their normal counterparts. In all cases, the reduced levels of hmC in tumor tissues were associated with a decrease in the relative expression of all three *TET* genes when compared with their matched normal tissues [13]. To shed light on the earlier stages of carcinogenesis, a pioneering study demonstrated a significant correlation between changes in the three epigenetic components in a rat model of estrogen-induced breast carcinogenesis [14]. Subsequently, the role of polycomb proteins as epigenetic silencers was shown in preneoplastic states in the pancreas of mice and rats [15], while other epigenetic alterations were documented during early stages of hepatocarcinogenesis in rats [16].

To date, the exploration of epigenetic changes, and their connection with other molecular events associated with the different steps from early preneoplastic lesions to malignant transformation and the acquisition of invasive properties, have not as yet been documented. In this study, the experimental approach used was based on, firstly, the characterization of a new collection of both neoplastic and preneoplastic mesothelial cells, established from an inbred strain of rats induced with asbestos, representing different stages in the tumorigenesis process. Secondly, among the preneoplastic cell lines, different groups and subgroups were identified according to the expression profiles of markers. This approach specifically revealed new findings related to the involvement of the relative expression of *TETs* and *Dnmts* in relation to the 5-hmC level, in the context of malignant transformation and the acquisition of metastatic potential, both in rat and human mesothelioma cells.

## RESULTS

### Rat mesothelial cell lines can be distinguished in two main categories: preneoplastic and neoplastic

**Figure 1:**
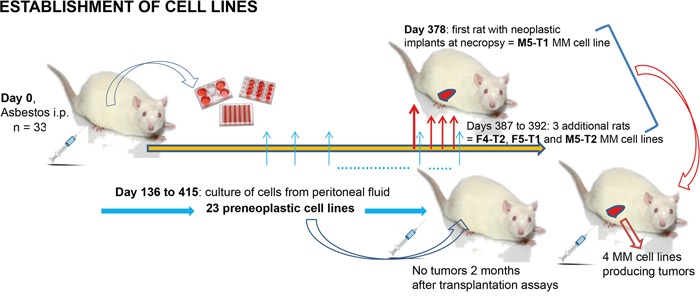
Establishment of the preneoplastic and neoplastic cell lines in F344 rats Scheme of establishment of the 27 cell lines and the four preclinical MM models.

**Figure 2:**
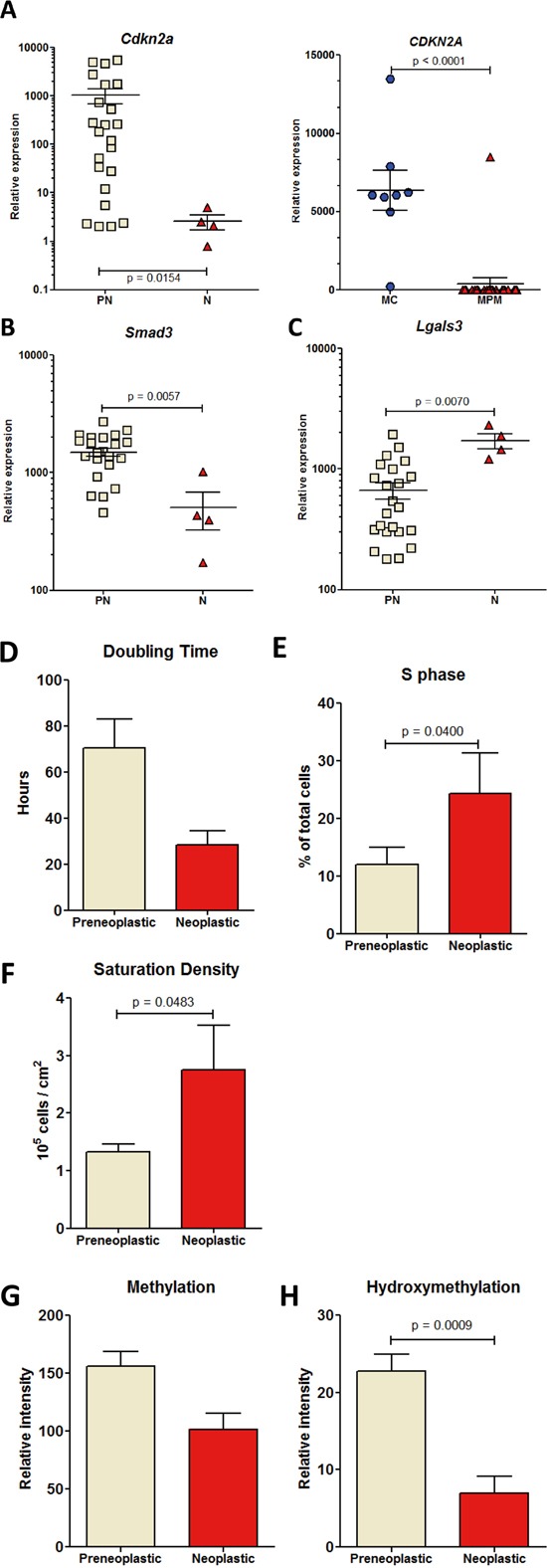
RT-PCR analysis of the relative expression of genes discriminating preneoplastic and neoplastic rat mesothelial cell lines **A.** *Cdkn2a* (*p 16*), in the rat experimental biocollection of cell lines (left) and the human biocollection of cell lines (right). MC, normal mesothelial cells; MPM, malignant pleural mesothelioma cells. **B.** *Smad3* and **C.** *Lgals3*. Growth characteristics of the rat cell lines in culture, mean doubling time **D.** proportion of cells in S phase **E.**, and mean saturation density **F**. Levels of methylation **G.** and hydroxymethylation **H.** determined by dot blot. Signals were quantified for each subgroup of cell lines and normalized to the intensity of the corresponding gel red signal.

Cell lines were initially distinguished as preneoplastic (“PN”, n = 23) or neoplastic (“N”, n = 4) according to: observations at necropsy on the individual rats from which each cell line was established, cell morphology in culture, and propensity or not to produce tumors 2 months after orthotopic transplantation of 5 × 10^6^ cells to syngeneic rats (Figure 1A). This discrimination was further confirmed by the analysis of expression profiles, growth patterns, and determination of the levels of cytosine methylation and hydroxymethylation. Analysis of *Cdkn2a* gene mRNA levels by qRT PCR revealed a significantly decreased relative expression in neoplastic relative to preneoplastic rat cell lines (Figure 2A, left). In human cell lines, the expression of *Cdkn2a* was also considerably decreased in pleural mesothelioma (MPM) relative to normal mesothelial cells (MC) (Figure 2A, right). A very significant decrease in the relative expression of *Smad3* and increase in the relative expression of *Lgals3* was also observed in neoplastic relative to preneoplastic rat cell lines (Figures 2B and 2C). Overall, compared with preneoplastic cell lines, neoplastic cells lines were characterized by a shorter mean doubling time (Figure 2D and Table S1), a higher proportion of cells in S phase (Figure 2E) and a higher saturation density (Figure 2F and Table S1). Cell migration analysis by scratching test did not reveal any difference between categories and groups of cell lines (Figure S1). As many solid malignant tumors show a dramatic decrease in their DNA methylation level relative to normal tisues, we analysed by dot blot the global methylation level in the two categories of cell lines and found that the level of cytosine methylation did not differ significantly between preneoplastic and neoplastic cell lines (Figure 2G). However, a very significant difference in the level of hydroxymethylation was observed, revealing an implication of this parameter during the tumorigenic process (Figure 2H).

### Among preneoplastic cell lines, two main groups were identified according to the expression profiles of eight genes and cell morphology in culture

**Figure 3:**
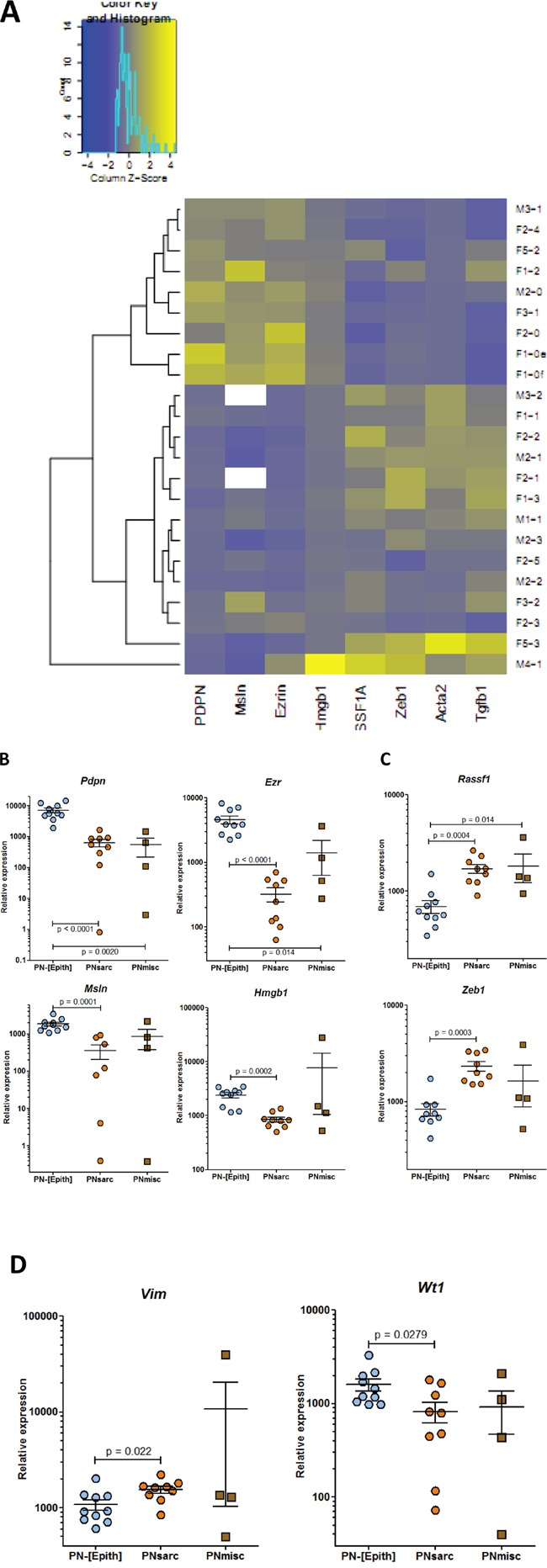
RT-PCR analysis of the expression of genes discriminating the different groups of preneoplastic rat mesothelial cell lines **A.** Heatmap of expression profiles of the preneoplastic rat cell lines. Genes coding for **B.** podoplanin (*Pdpn*), ezrin (*Ezr*), mesothelin (*Msln*), HMGB1 (*Hmgb1*); **C.** RASSF1A (*Rassf1*), ZEB1 (*Zeb1*); **D.** vimentin (*Vim*), and WT1 (*Wt1*).

**Figure 4:**
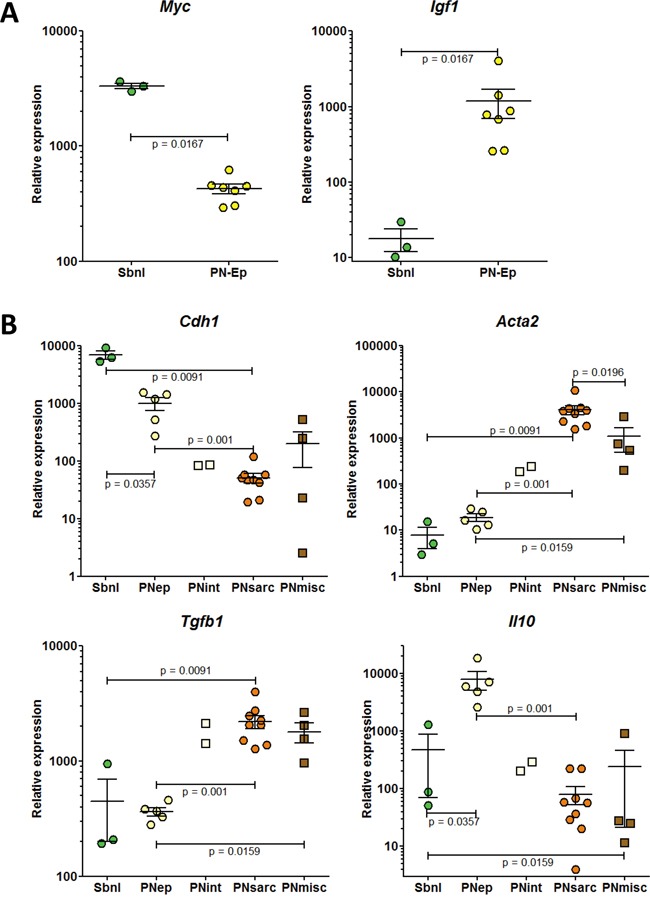
RT-PCR analysis of the expression of genes discriminating the different subgroups of epithelioid preneoplastic rat mesothelial cell lines Genes coding for **A.** c-Myc (*Myc*) and IGF-1 (*Igf1*); **B.** E-cadherin (*Cdh1*), α-smooth muscle actin (α-SMA) (*Acta2*), TGFβ (*Tgfb1*), and IL-10 (*Il10*).

The 23 preneoplastic cell lines comprised two main groups, differing in their general “epithelioid” (named PN-[Epith]) and “sarcomatoid” (named PNsarc) morphology in culture (Figure S2). This observation was associated with differences in the expression profiles of 8 genes (Figure 3A). Analysis of mRNA levels revealed a highly significant decreased expression of genes coding for podoplanin, ezrin, mesothelin, and Hmgb1 (Figures 3A, 3B), and increased expression of genes coding for Rassf1a and Zeb1 in the PNsarc group relative to the PN-[Epith] group (Figure 3A, 3C). These two main groups also differed by a significant increase and decrease in the relative expression of the genes coding for vimentin and WT-1, respectively (Figure 3D). The identification of the PNsarc group of 9 cell lines was confirmed by an increased expression of genes coding for the mesenchymal marker alpha smooth muscle actin (α-SMA), and TGFβ, an inducer of the epithelial-to-mesenchymal transition (EMT), relative to the 10 cell lines of the PN-[Epith]) group (Figure 4). Finally, a third group of 4 cell lines, which shared with PNsarc a low and high mean mRNA level for the genes coding for podoplanin and Rassf1a, respectively, presented intermediate expression levels for other genes (Figures 3A–3D). This group was also characterized by an important dispersion of mRNA levels for the ten genes evaluated, relative to the PNsarc group, and very different morphological characteristics in culture. For all these reasons, this group was defined as “miscellaneous” (named PNmisc).

### Among the preneoplastic cell lines with epithelioid morphology, three subgroups were identified according to the expression profiles of four additional genes

Among the preneoplastic cell lines with epithelial morphology, a first subgroup (named subnormal, “Subnl”) was identified by the highest expression of *c-Myc*, a proto-oncogene gene the deregulation of which contributes to deregulated DNA synthesis and genomic instability, and the lowest expression of *Igf1*, a gene involved in the deregulation of cellular energetics (Figure 4A). A second subgroup of cell lines (named PNep) differed from the former by a dramatic increase in the expression of *Igf1* and *Il10*, coding for an anti-inflammatory cytokine, and a reduction in the mean expression of *Cdh1*, coding for the epithelial marker, E-cadherin (Figure 4B). Finally, a third subgroup of two cell lines, named PNint, represented an intermediate situation between the PNep and PNsarc subgroups, based on the relative expression of *Cdh1*, *Acta2* and *Tgfb1* (Figure 4B).

### Characterization of the neoplastic cell lines and tumors

The four different neoplastic cell lines shared in common a significant decrease in the expression of *Ezr* and *Hmgb1*, compared with the PN-[Epith] group of preneoplastic cell lines, however their expression profile was comparable to that of the PNsarc group (Table S2). Among the four neoplastic cell lines, three, F4-T2, F5-T1 and M5-T1 differed by a lower expression of *Cdkn2a*, *Rassf1* and *wt1*, and a higher expression of *Msln*, *Pdpn*, *Acta2*, *Zeb1* and *Tgfb1*, compared with M5-T2, (Table S2), and a propensity to produce metastases in different normal tissues after orthotopic transplantation into syngeneic rats. The M5-T2 tumor differed from the three others by the absence of metastatic potential, with tumor cell development restricted to the omentum. *In vitro*, the M5-T2 cell line was also characterized by a low saturation density (Table S1). In contrast, the most aggressive neoplastic cell line, M5-T1, presented a specific growth pattern, characterized by the shortest doubling time, and the highest saturation density (Table S1). Cell cycle analyses revealed that this cell line showed the highest percentage of cells in S phase, the presence of a tetraploid population, and sphere forming capacity in culture (Figure S3). These observations contrasted with the proportion of cells in the different phases in the diploid F3-1 cell line, chosen as representative of the PNep subgroup of preneoplastic cell lines.

**Figure 5:**
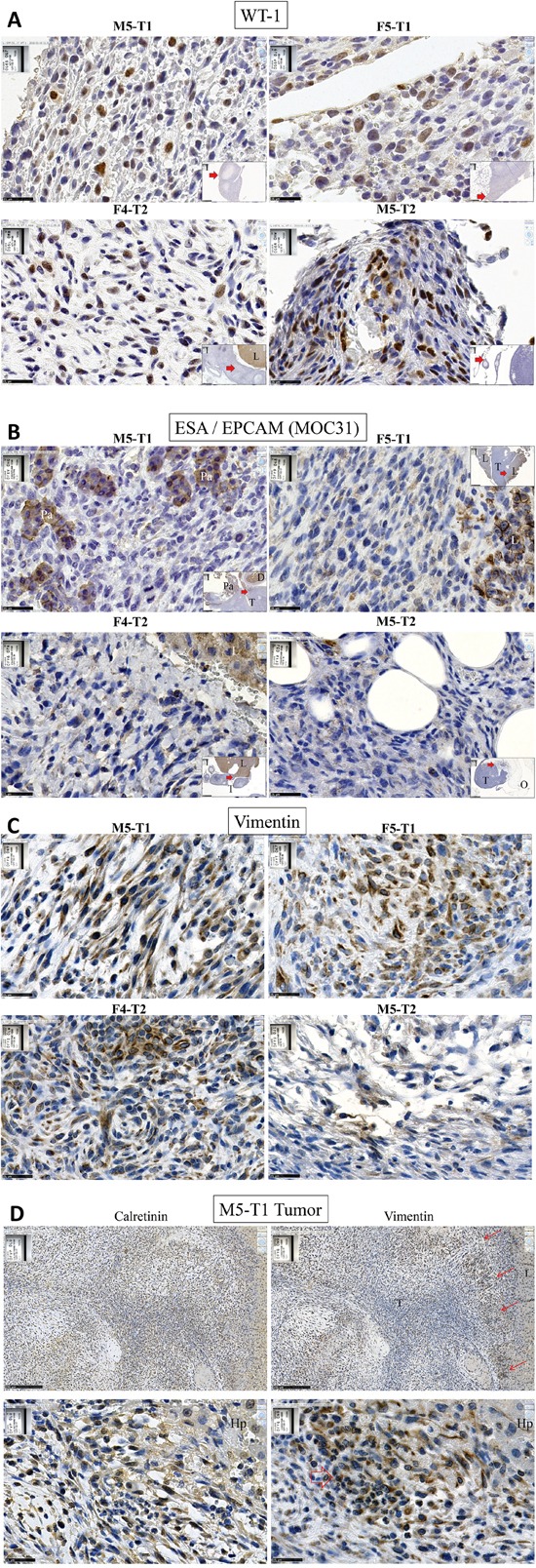
Immunohistochemical characterization of rat mesotheliomas Immunohistochemical staining for **A.** WT1; **B.** ESA/EPCAM (MOC 31); **C.** vimentin of the tumors collected from syngeneic F344 rats after i.p. injection of the neoplastic rat cell lines M5-T1, F5-T1, F4-T2 and M5-T2, x 800, the scale bars represent 25 μm. Inserts show general views, x25, the scale bars represent 1 mm and the red arrow indicate the location of the magnification. Pa, pancreas; T, tumor; D, duodenum; L, liver; O, omentum. **D.** Comparison of IHC staining for calretinin (left) and vimentin (right) in a liver metastasis of M5-T1. Top, x 100, the scale bars represent 250 μm; bottom, x 800, the scale bars represent 25 μm; red arrows indicate foci of tumor cells exhibiting strong positivity of vimentin at the tumor front. Hp, hepatocytes.

Characterization of the four tumor models by immunohistochemical markers revealed that, although the staining was moderate and heterogeneous, all tumor cells exhibited positivity for WT1, a common positive mesothelioma marker (Figure 5A). On the other hand, all tumor cells were negative for ESA/EPCAM/(MOC 31) (Figure 5B), a negative mesothelioma marker commonly used to distinguish mesotheliomas from carcinomas. Comparison between immunostaining for calretinin and vimentin in a liver metastasis of M5-T1, the most aggressive tumor, revealed a contrast between a moderate, overall homogeneous positivity of tumor cells for calretinin, and the presence of foci of cells strongly positive for vimentin close to the tumor front (Figure 5C).

To characterize the chromosomal events potentially associated with the molecular changes, complete spectral karyotypes and karyotypic formulas were analyzed for M5-T1 in comparison with the F3-1 cell line (Figure S4 and Table S3). The unique structural rearrangement observed in the F3-1 cell line was a der(15)t(1;15)(q?41 ;q2?2), leading to a distal gain of chromosome 1 in 15% of metaphases (Figure S4A, D). Fifty-nine percent of F3-1 metaphases displayed a normal karyotype (42, XX) and 21% of metaphases had a modal chromosome number of 41. Compared with the F3-1 cell line, the study of ploidy in M5-T1 cells revealed the presence of two subpopulations in accordance with cell cycle analysis by flow cytometry (Figure S3). A near-diploid population representing about 61% of the total metaphases analyzed had a modal chromosome number of 43. The tetraploid population represented about 39% of the total metaphases and had a modal chromosome number of 86. The M5-T1 near-diploid population retained the structural abnormality der(15)t(1;15) in two metaphases only, but, interestingly, chromosome 15 was also found to be fused to chromosome 2, and once with chromosome 4, in the tetraploid metaphases with the same breakpoint on chromosome 15 (Figure 4D). Otherwise, no more metaphases with normal karyotypes were observed and all the chromosomes except chromosomes 7 and 16 were involved in either numerical alterations and/or structural alterations such as translocations and deletions. The major recurrent alteration was deletion of chromosome 5 (82% of all M5-T1 metaphases).

### Changes in the expression of epigenetic regulators and 5-hmC levels in rat and human mesothelial cell lines and mesotheliomas

During the past decade, a number of studies investigating the role of epigenetics in cancer development have revealed altered hmC levels and/or *TET* and/or *DNMT* gene expression in human tumor tissues relative to their normal counterparts [9–13]. We previously showed a significant decrease in the global level of the 5-hydroxymethylcytosine, but not 5-methylcytosine between preneoplastic and neoplastic cell lines (Figure 2G, 2H). To confirm this both in rat and human MM cell lines, we investigated the changes in the expression of all these epigenetic regulators and tried to shed light in parallel on events occurring in preneoplastic mesothelial cells, an element that has never been documented.

**Figure 6:**
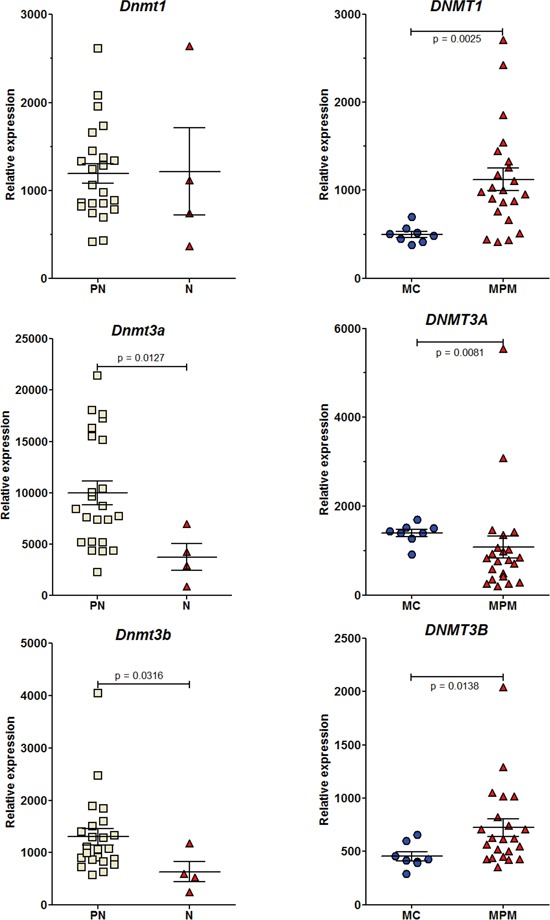
RT-PCR analysis of the relative expression of the three DNA methyltransferase genes Rat (left column) and human (right column) cell lines.

**Figure 7:**
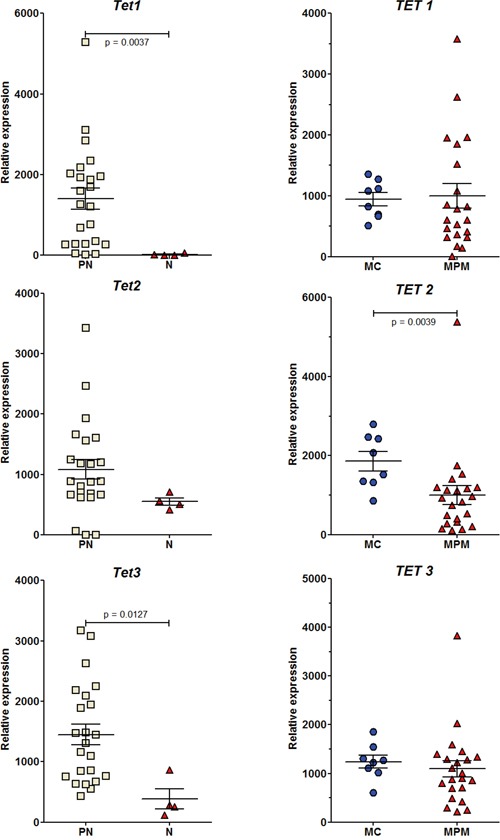
RT-PCR analysis of the relative expression of the three Ten-Eleven Translocation (TET) dioxygenase genes Rat (left column) and human (right column) cell lines.

*Dnmt3a* expression showed a significant decrease in neoplastic compared to preneoplastic cells in both rat and human mesothelial cell lines (Figure 6). A significant decrease in the expression of *Dnmt3b* was also observed in the rat. In parallel, a decrease in the expression of all *TET* genes was observed in both species (Figure 7). However, this decrease was significant in the rat for *TET1* and *TET3* between preneoplastic and neoplastic cell lines (Figure 7), and in humans for *TET2* between normal mesothelial cells (MC) and malignant pleural mesothelioma cell lines (MPM) (Figure 7). Of the four neoplastic rat cell lines, M5-T1 also presented the lowest expression of *TET3* (117.5 vs. 255.5, 287.3, and 863.9 for the three others). Finally, among preneoplastic rat cell lines, a significant decrease in the expression of *Dnmt3b* and all *TET* genes was observed in the three invasive neoplastic cell lines, relative to the PNsarc group (Figure S5).

**Figure 8:**
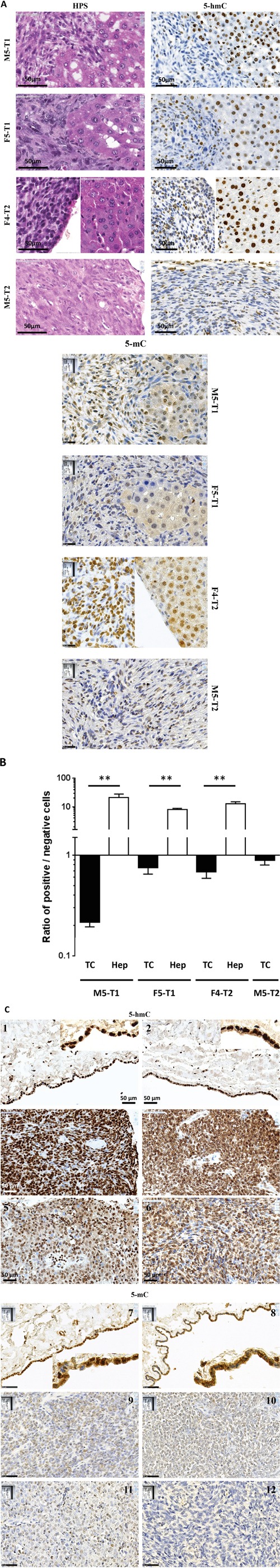
Immunohistochemical staining for 5-hmC of rat and human mesotheliomas **A.** Histology and immunohistology of rat mesotheliomas collected three weeks after i.p. inoculation of the four neoplastic cell lines into syngeneic F344 rats. Left column: HPS staining, detailed views of the fields denoted by the red arrows on general views (see Figure S5), showing the morphology of tumor cells invading the liver tissue (M5-T1, F5-T1), attached to it (F4-T2) or growing on the omentum (M5-T2) without metastases in the vicinal normal tissues. The scale bars represent 50 μm. Middle column: IHC figures of anti-5-hmC immunostaining of the tumor tissue (left part) and liver parenchyma (right part), the scale bars represent 50 μm. Right column: IHC figures of anti-5-mC immunostaining of the tumor tissue (left part) and liver parenchyma (right part), the scale bars represent 25 μm. **B.** Semi-quantification of 5-hmC immunostaining in the four rat mesothelioma models. The numbers of positive and negative cells in the tumor tissue and the liver parenchyma were determined on five high-magnification fields (x 800) containing both tumor tissue and liver parenchyma (except for the noninvasive M5-T2 cell line). **C.** Representative images of normal mesothelial cells of the pleura and pericardium (1, 2, inserts show high magnification), and malignant mesothelioma, grade I (3, 4) and grade II (5, 6), stained with an anti-5-hmC antibody, scale bars represent 50 μm. IHC of normal mesothelial cells of the pleura and pericardium (7, 8, inserts show high magnification), and malignant mesothelioma, grade I (9, 10) and grade II (11, 12), stained with an anti-5-mC antibody, scale bars represent 50 μm.

Representative images of the M5-T1, F5-T1, F4-T2 and M5-T2 tumors (HPS staining of detailed views in Figure 8A, left column, general views in Figure S6) globally reveal low levels of staining for 5-hmC, which contrasts with the strong nuclear signals observed in the vicinal normal liver tissue (Figure 8A, right column). Conversely, in agreement with methylation levels on dot blots (Figure 2G), immunohistochemistry for 5-mC on the same sections showed equivalent levels of staining in tumor and normal liver. Quantification of the staining intensity for 5-hmC in tumor vs. liver revealed marked differences between the four different tumor types, with M5-T1 exhibiting the highest ratio (Figure 8B).

Immunohistochemical analysis of 5-hmC levels in human tissue microarrays revealed a progressive fall in the mean intensity of positive staining between normal mesothelial cells, grade I mesothelioma cells, and grade II mesothelioma cells. In parallel, the proportion of positively stained cells decreased, while the proportion of negative cells (in blue) increased (Figure 8C).

## DISCUSSION

During development, the mesothelium, which consists of a monolayer of specialized cells with a typical cobblestone-like morphology, retains its potential to differentiate into cells of different phenotypes [17]. This mesenchymal “pluripotent” origin of mesothelioma cells is suggested to be responsible for the complex biology of MM and for the lack of efficacy of current therapies [2]. When injured through prolonged contact with fibers such as asbestos, mesothelial cells are transformed, move into the serosal fluid and thus can be easily isolated, cultured, characterized, and for some of them, transplanted into laboratory rodents to produce tumors. In this study, we took advantage of this diversity of experimental situations to establish a collection of cell lines which could significantly contribute to understanding some of the mechanisms of mesothelioma tumorigenesis.

### Expression profiles of three genes discriminate all neoplastic from preneoplastic rat mesothelial cell lines

Among this biocollection, four cell lines shared in common the potential to generate tumors *in vivo* after orthotopic transplantation into the peritoneal cavity of syngeneic rats. Our finding that *Lgals3* was up-regulated at the mRNA level in neoplastic relative to preneoplastic cell lines is in good agreement with accumulating evidence indicating that galectin-3 is closely involved in tumor cell transformation, migration, invasion and metastasis for a number of cancers [18, 19]. In addition, the observation of a higher expression level of *Lgals3* in the three invasive neoplastic cell lines compared with the non invasive M5-T2 suggests a relation with the tumor grading, as previously demonstrated in human gliomas [20].

*Cdkn2a* also represents a tumor suppressor gene that has previously been reported to represent a target for biological studies on mesothelioma [1, 21]. Investigations on this gene have proven to be a reliable way of separating benign from malignant mesothelial proliferations in tissues [22], in particular for the sarcomatoid subtype [23, 24]. In this study, the expression of *Cdkn2a* (*p16*) was significantly reduced, both in neoplastic relative to preneoplastic cell lines, and in human pleural mesothelioma relative to normal human mesothelial cells, thus confirming the potential of this gene for the diagnosis of MM. *Smad3*, a gene for which the expression level was dramatically reduced in a tumorigenic cell line transformed with activated H-Ras compared with the normal parental epithelial cells [25], recently appeared to represent an important determinant of the progression of tumorigenesis. Herein, the significant decrease in the expression of *Smad3* observed in all neoplastic relative to preneoplastic cell lines is also in good agreement with the initial report of Han and colleagues, demonstrating that suppression of Smad3 expression occurred in human gastric tumor cells in contrast to neighboring normal tissue [26].

### Characterization of preneoplastic rat mesothelial cell lines

To date, most studies on preneoplastic cell lines have been conducted either on spontaneously transformed normal cells in culture [27] or on immortalized cell clones isolated from biopsies [28]. Additional insights have been obtained from histopathological observations made on both preneoplastic and neoplastic cells [29] and by the use of transgenic animals [30]. However, all these experimental approaches present important limitations. Cell lines selected under *in vitro* conditions for a long period of time present many alterations of specific cellular pathways compared with tissues [31]. Data obtained from biopsies or/and histopathologic observations from patients are limited by the genetic heterogeneity of the source materials. Finally, many inbred transgenic and targeted mutant mice have been created using animals of mixed genetic backgrounds [32]. In contrast with all these situations, in our study, the biocollection of cell lines was obtained from F344 rats, an inbred strain known for its stable genetic background [33], induced with the same material at the same age, which is likely to significantly limit the number of parameters involved in mesothelioma tumorigenesis.

Among the 23 preneoplastic rat mesothelial cell lines established, two groups were identified according to the expression profiles of eight genes and to morphology in culture. The decreased expression of the *Msln* gene observed between the PN-[Epith] and PNsarc groups is in good agreement with the differences in the expression profiles previously observed at both the mRNA and protein levels [34], and with immunohistochemical findings reported by Ordóñez [35]. The low level of *Msln* expression in the PNsarc group, in contrast to the PN-[Epith] group, could also be due to instability of *Msln* mRNA, according to the presence of a polymorphic variant and regulation by micro-RNA [36]. Similarly, although podoplanin has been presented as an interesting immunohistochemical marker in the diagnosis of mesothelioma [37], its expression is frequent in the epithelioid subtype but usually absent in the sarcomatoid subtype [38, 39], in agreement with our findings on PNsarc cell lines. This observation was further confirmed in iron-induced high-grade rat MM where only epithelioid subtypes were strongly positive for podoplanin [21].

We found a decrease in the expression of the *Ezr* gene, coding for ezrin, a multifunctional membrane cytoskeleton linker that regulates the structure and function of specific domains of the cell cortex. This could be explained by the fact that few microvilli have been observed in the sarcomatoid subtype [40], as ezrin was originally identified in high amounts in these structures [41], an ultrastructural characteristic of normal mesothelial cells and epithelioid MM [42]. Regarding *Hmgb1* expression, besides its well-documented proinflammatory role, evidence for its restorative effects leading to tissue repair and regeneration have previously been reported [43]. Thus, the decrease in its expression observed both in PNsarc and neoplastic cell lines could participate in nuclear instability, in good agreement with the fact that its localization in the nucleus maintains nuclear stability under stress [44].

### Changes among preneoplastic epithelioid rat mesothelial cell lines

Among PN-[Epith] cell lines, the first significant *Cdh1* expression loss occurred between the Sbnl and PNep subgroups, preceding the increase in expression of both *Tgfβ1* and *Acta2* in the two PNint cell lines, suggesting a link with the EMT process [45]. In parallel, the observation of a dramatic increase *Igf1* expression also agrees with the recent demonstration of the role of deregulation of cellular energetics in the development of preneoplastic lesions [7], in relation with a very early event of cadherin modulation in the oncogenic EMT program [46]. Under certain conditions of stimulation, the increased production of IL-10 transcript by rat peritoneal mesothelial cells has also been shown [47], suggesting a relation with an inflammatory process. The dramatic change observed in the expression of *Myc* suggests a link with the dynamic process of genomic instability related to this oncogene, which has been extensively documented during the past two decades [48]. Finally, the elevated expression of *Tgfb1*, *Zeb1* and *Acta2* and the additional loss of expression of *Cdh1* also observed between the PNep subgroup and the PNsarc group are consistent with the dual role that has been reported for *Zeb1*, a repressor of gene transcription with a target gene such as *Cdh1*, and also a transcriptional activator that targets genes such as *Acta2* [49].

### Expression profiles and IHC markers of invasive MM rat cell lines share more homology with the PNsarc group than does M5-T2, suggesting a mesenchymal phenotype

Mesothelioma tumorigenesis involves mesothelial progenitor cells [17, 50, 51] able to switch between different cell phenotypes in both directions depending on the local environment, which can differentiate to epithelioid or mesenchymal phenotypes. We previously showed that among the four mesothelioma cell lines, three exhibited invasive capacities in the Boyden test, in agreement with their metastatic potential observed in this study [52]. Herein, the dramatic decrease in the expression of *Pdpn*, *Ezr*, *Msln*, *Hmgb1*, *Wt1* and *Cdh1* shared by these three cell lines and the PNsarc group, compared with the PN-[Epith] group, tend to confirm that these three cell lines and the PNsarc group present in common the characteristics of a mesenchymal phenotype. In agreement with previous findings [40], the overall weak expression of calretinin and clear positivity for vimentin of tumors generated by these three MM cell lines leads to the conclusion that these tumors can be diagnosed as sarcomatoid.

In contrast, in the M5-T2 cell line, this common decrease was restricted to *Ezr*, *Msln*, *Hmgb1*, and *Cdh1*, the high expression profiles of two genes, *Pdpn* and *Wt1*, together with the higher percentage of cells strongly positive for WT-1 in IHC suggesting the occurrence of some reminiscent homologies with the PN-[Epith] group. The singular situation of the M5-T2 cell line was reinforced by the observation of a maximum expression of five other genes, *Rassf1*, *Zeb1*, *Vim*, *Acta2* and *Tgfb1*, which was common to the PNsarc group, in contrast with the three invasive mesothelioma cell lines which presented significantly lower expression levels.

### Karyotype alterations and aggressiveness

The comparison of expression profiles with the results of karyotype analysis for the M5-T1 cell line allows the definition of several points of interest that appear to be related to aggressiveness. A recent study confirmed that *p16* deletion is a good marker of malignancy, both in pleural and peritoneal mesotheliomas with invasive components [53]. A common chromosomal deletion was detected at 5q32, containing the *Cdkn2a* gene, and a homozygous deletion was observed in the majority of cases from epithelioid to sarcomatoid and biphasic mesotheliomas [54]. The results from our model tend to confirm this fact, as M5-T1 cells exhibited the lowest level of *Cdkn2a* expression while spectral karyotyping of this cell line revealed a recurrent, large deletion of one rat chromosome 5, including the q32 band, in 82% of metaphases, suggesting at least loss of heterozygosity for *Cdkn2a*. In the rat, the *Pdpn* gene is also localized very close to *Cdkn2a* on chromosome 5, at 5q36, where this deletion was observed, a point that could also explain the lowest *Pdpn* expression level of this cell line. The dramatic decrease observed in the expression of *Smad3* could also be related to the important deletion of chromosome 8, which bears this gene. Finally, the very low expression level of *Msln* specifically observed in the three invasive cell lines, in contrast to M5-T2, also appears to be related to another deletion occurring on chromosome 10, this the *Msln* gene being localized to 10q12-q21 inthe rat.

### Epigenetic regulators and tumorigenesis

Global DNA hypomethylation and locus-specific DNA hypermethylation have been identified as key features of many cancers [55, 56]. In the specific case of mesothelioma, epigenetic dysregulation is now established to be a critical mode of action of asbestos, and it has been suggested to be associated with inflammation-related processes common to several cancers. A large study of epigenomic alterations in 158 pleural MM samples obtained from human patients revealed hundreds of loci that had different methylation levels between tumor and non tumor pleura, identifying seven distinct methylation subclasses and leading to the idea that asbestos-associated inflammation could drive this epigenetic diversity [57]. In the latter study, *Dnmt3b* was mentioned in the list of genes having significantly lower methylation in tumors, suggesting epigenetic dysregulation. This observation presents interesting connections with our findings in the rat biocollection of cell lines, a significant decrease in the expression of *Dnmt3b* being observed between all preneoplastic and neoplastic cell lines, as well as between the PNsarc group and neoplastic cell lines. The different evolution observed in the human biocollection for the expression of this enzyme, while a common pattern of expression profile was observed in the two species for *Dnmt3a*, could also be explained by recent reports indicating that the two enzymes have overlapping functions and that their roles in cancers may be more complex than previously believed [58]. In our study, the final fall in expression of *de novo Dnmt3a* and *Dnmt3b* could be explained by the recent demonstration of downregulation after induction of EMT by TGFβ in lung cancer cells [59]. Our results are also in good agreement with the recent finding that both *de novo* DNMTs showed a different pattern of expression relative to DNMT1 during carcinogenesis, with an overexpression in early cancer stages and a final reduced expression in tumors [60]. Thus, this event could represent the situation found in MM with the worst outcome. The involvement of DNA methylation in EMT has also been documented, hypermethylation of the promoter region being one of the key factors leading to a reduced mRNA expression of *Cdh1* during EMT [61]. This could contribute to the final fall in the expression of *Cdh1*.

In our study, another striking result concerned the parallel evolution of the expression of *TET2*/*TET3* and *Dnmt3a*/*Dnmt3b*. So far, although *TET3* has been considered to be the most important regulator [62], relationships between *TET3* and cancer have been poorly investigated. A recent study demonstrated that the low level of 5-hmC observed in carcinoma *in situ* cells (CIS) of the testis corresponded to the absence of expression of TETs in these cells, especially *TET3* [63]. We observed here that the most aggressive MM cell line, M5-T1, showed the lowest expression of *TET3*, an observation that could be explained by the deletion of the q34-q44 region of chromosome 4, which includes the *TET3* gene. In addition to *Cdkn2a* on chromosome 5, another observation relating karyotypic alterations to aggressiveness concerns the *NF2* gene coding for merlin, the second major alteration in human MM, which was shown to be the target tumor suppressor gene of 22q12 loss and found to be mutated in the majority of human MM [64]. Interestingly, this gene, located on rat chromosome 14q22, was included in a deletion observed in 79% of M5-T1 metaphases.

The parallel lowest expression of *Dnmt3b* and lowest 5-hmC level, especially in the tumor cells located at the invading front in the liver tissue, provides the first evidence of a clear link between these three epigenetic parameters. Evidence of collaboration between TET2 and TET3 proteins to suppress aberrant hematopoiesis and hematopoietic transformation has also been reported recently [65]. Interestingly, the parallel between *DNMT3A* and *TET2*, and their mechanisms of cooperation, have been mentioned for future studies [12]. *TET2* represents a critical regulator for normal and malignant hematopoiesis [66], and its expression has recently been shown to represent a prognostic factor for colorectal carcinoma recurrence and outcome [67]. *TET2* expression is also significantly associated with 5-hmC levels in esophageal squamous cell carcinoma [68].

In conclusion, in this work we present a biocollection of rat mesothelial cells at different stages of carcinogenesis. The transcriptomic and karyotypic characterizations of these rat MM cells showed high similarities with human MM cells, highlighting the pertinence of our preclinical models for future evaluation of new therapeutic strategies. Finally, evaluation of this biocollection allowed us to suggest that epigenetic alterations, downregulation of TETs and DNMTs and a decrease of 5-hmC in tumor cells are all correlated with the acquisition of an aggressive phenotype, in good agreement with human MM.

## MATERIALS AND METHODS

### Establishment of experimental rat cell lines

Fischer F344 rats were obtained from Charles River Laboratories (L'Arbresle, 69, France) and maintained under standard conditions in the UTE IRS-UN animal holding area in agreement with European Union guidelines for the care and use of laboratory animals in research protocols. The experiments were approved by the regional ethical committee for animal experiments (CEEA.2011.38). Rats were fed a pelleted standard diet (RM1, Special Diet Services, Witham, Essex, UK), with tap water *ad libitum*, and were anesthetized via an isoflurane chamber (Forene®, Abbott France) and euthanized with Dolethal® (Centravet, Pluduno, Plancoët, France).

The cell lines used in this study belong to a biocollection established in 2011 [http://www.inserm-transfert.fr/]. To establish this biocollection, at 8 weeks of age, a group of 33 rats (16 males and 17 females) received an intraperitoneal inoculation of 10 mg crocidolite fibers suspended in 0.5 ml 0.9% NaCl (UICC analytical sample, ref. 02704A, Neyco, 75017 Paris, France). Between 136 and 415 days after induction, after euthanasia, 2 ml sterile RPMI medium was injected intraperitoneally, the peritoneal lavage fluids were collected under sterile conditions and then placed into culture. This step led to the establishment of 23 transformed mesothelial cell lines. After 378 days of induction, one male rat was necropsied about one hour after death, presenting signs of hemorrhage, widespread neoplastic implants and nodules in the peritoneal cavity as previously described [69], which, when dissociated with a scalpel and cultured, led to the first neoplastic MM cell line M5-T1. During the next two weeks, three additional MM cell lines were established from dissociated neoplastic implants and nodules were collected on two females and one male euthanized rats (F4-T2, F5-T1 and M5-T2). These cell lines were distinguished from the former 23 by their capacity to produce macroscopic tumors in syngeneic F344 rats three weeks after intraperitoneal inoculation of 5 × 10^6^ cells.

### Human cell lines

Mesothelioma cell lines were established from pleural effusions collected by thoracocentesis of patients with cancers, as previously described [70]. All patients provided written informed consent and the study was approved by the ethics committee “Comité de Protection des Personnes Ouest IV – Nantes, CPP N° 877/2011”, CHU de Nantes, France. Isolation and culture of normal mesothelial cells from pleura have been described previously [70]. Primary peritoneal mesothelial cells (PMC) were purchased from Tebu-bio biosciences and cultured according to the manufacturer's recommendations.

### Cell culture and chemicals

All cell lines were maintained in RPMI 1640 medium (Invitrogen) supplemented with 10% heat-inactivated fetal calf serum (Eurobio), 100 U/ml penicillin, 0.1 mg/ml streptomycin, 2 mM L-Glutamine (Sigma-Aldrich, St Louis, MO, USA) and cultured at low passage level (6 to 10) in a humidified atmosphere of 5% CO_2_ at 37°C.

### Proliferation assays and cell cycle analysis

For determination of the population doubling time in the mid-log phase and the saturation density at plateau, in order to approach the conditions of proliferation *in vivo* after transplantation, cell lines were seeded at 3 × 10^5^ cells/well at time 0 in 6-well plates (Nunclon delta, Nunc AS, Denmark) and counted in a Malassez hemocytometer, the entire growth curve being obtained from at least 12 time points. Cell cycle analysis was conducted on a NucleoCounter® NC-3000™ (ChemoMetec, Allerød, Denmark) using NC-Slide A8™ after trypsinization, centrifugation, fixation of cells with ethanol, and incubation with DAPI at 37°C for 5 minutes. The cell number in each phase of the cell cycle was determined and calculated as a percentage of the total cell population.

### Measurement of cell migration

Cell migration was analyzed for all rat cell lines by the scratching test [71]. When cell density was confluent, for each cell line cultivated in 12-well plates (3.5 cm^2^ / well), one wound line in the form of a cross was made by scratching the cell monolayer with a plastic pipette tip. Floating cells were washed out and fresh medium was added. The narrowing width of the scratch was recorded by taking photographs under an inverted Zeiss Axio Observer.Z1 microscope from 17 h after the scratch.

### Total RNA isolation and real-time PCR

Total RNA was extracted from one 75 cm^2^ culture plate at preconfluence for each cell line using the NucleoSpin RNA II Kit according to the manufacturer's instructions (Macherey-Nagel, Hoerdt, France). The total RNAs were next treated with an rDNase solution to remove contaminating genomic DNA, and subsequently purified. One microgram of total RNA was reverse-transcribed using Moloney-Murine Leukemia Virus Reverse-Transcriptase (Invitrogen). PCR reactions were performed using an Mx4000 thermocycler (Stratagene) with 10X QuantiTect Primer Assays (Qiagen), and the RT^2^ Real-Time SYBR-Green/ROX PCR Mastermix (tebu-bio, Le Perray-en-Yvelines, France), according to the manufacturer's instructions. The thermal cycling protocol was followed by a melting curve analysis to check for the absence of nonspecific products. For each transcript, the efficiency of the PCR reaction was determined by the slope of the standard curve generated from serial dilutions of the same cDNA sample (pool of reverse-transcribed RNA samples). The relative amount of target RNA, called the Starting Quantity (SQ), was determined using the MxPro software, by comparison with the corresponding standard curve for each sample performed in duplicate. Each transcript level was normalized by division with the expression values of the acidic ribosomal phosphoprotein P0 housekeeping gene (*RPLP0*), used as an internal standard.

### Tumor samples and immunohistochemistry

For histological examination, the paraformaldehyde-fixed, paraffin-embedded sections of rat tumor samples (M5-T1, F5-T1, F4-T2 and M5-T2), and surrounding normal/invaded tissues when present, were cut with a Bond Max automaton (Menarini, Rungis, France) and stained with hematoxylin-phloxine-saffron (HPS). A human mesothelioma array with normal mesothelium tissue (lung and cardiac pericardium) was obtained from US Biomax Inc. (Rockville, MD, USA). Antibodies used for immunohistochemical analyses were anti-WT-1 ab15249, anti-calretinin ab16694 and anti-vimentin ab8978 from Abcam, anti ESA/EPCAM (MOC 31) LS-C331328 from LSBio, and anti-5-hmC 39769 (1/200) and anti-5-mC 61255 (1/60) from Active Motif Europe (La Hulpe, Belgium), with an anti-mouse secondary antibody and the N-Histofine Simple Stain Mouse MAX Peroxidase (Nichirei Biosciences, Tokyo, Japan) as the detection reagent. Histopathology slides were scanned with a Nanozoomer 2.0 HT (Hamamatsu, Japan). To semiquantify histochemical staining on rat tumor slices, five randomly selected fields of 27,000 μm^2^ were analyzed both by manual counting and by color deconvolution of TIF images using Fiji software and the Otsu method [72]. Histochemical staining of the human mesothelioma array with anti-5-hmC and anti-5-mC was analyzed on 20 samples of normal tissue, 20 samples of grade I malignant mesothelioma (with tumor invading the submucosa) and 16 samples of grade II malignant mesothelioma (with tumor invading the muscularis propria). The small number of samples available on the microarray did not allow an analysis of grades III-IV.

### Dot blots

DNA samples were denatured by the addition of denaturation buffer (0.1 mM NaOH) and subsequently heated for 5 minutes at 95°C. The reaction was then stopped by addition of 6.6 mM ammonium acetate. Samples were rapidly chilled for 5 minutes on wet ice and then applied to a positively charged nylon membrane (Immobilon-P Transfer, Millipore), previously charged and rehydrated by dipping for 15 seconds into ethanol and 5 minutes in tris-buffered saline with Tween 20 (TBST). The membrane was then air dried for 15 minutes. Denatured DNA was spotted onto the membrane and air dried for 45 to 60 minutes, UV-cross-linked to the membrane (UV Stratalinker 2400), and incubated for 1 hour with saturation buffer (TBST 1%BSA). Primary antibody (5-hmC dilution 1/10,000; 5-mc dilution 1/5,000, Active Motif, La Hulpe, Belgium) was incubated overnight at 4°C with agitation. The membrane was washed three times with TBST and incubated with the anti-rabbit secondary antibody (HRPanti IgG, Covalab). The membrane was washed again three times with TBST and the signal was revealed using enhanced chemiluminescence (ECL) substrate (Immobilon western chemiluminescent HRP substrate, Millipore) and read with a Biorad Chemidoc MP Imaging System. To control the DNA load, DNA was quantified using gel red and the membrane was scanned with the Biorad imager. Dot blot signals were quantified and normalized to the intensity of the corresponding gel red signal. To ensure specificity of the antibody, naked DNA, methylated DNA, and 5-hmC DNA control (Active Motif) were used (data not shown).

### Multicolor FISH (M-FISH)

Metaphases were obtained using standardized harvesting protocols as described previously. Briefly, colcemid solution (0.03 μg/ml) was added to F3-1 and M5-T1 cells 90 minutes and 30 minutes before harvesting, respectively. Cells were treated with hypotonic solution (0.075M KCl), fixed three times with Carnoy's fixative (3:1 methanol/acetic acid) and spread on prechilled glass slides. M-FISH was performed with the aim of identifying numerical and structural alterations in both cell lines. The probe cocktail containing differentially labeled, chromosome-specific painting probes – except for chromosomes 13 and 14, which were distinguished by their DAPI banding (22XRat kit MetaSystems, Altlussheim, Germany) – was denatured and hybridized to denatured metaphase spreads according to the manufacturer's protocol for the Multicolor FISH probe kit for rat chromosomes (MetaSystems). After counterstaining with DAPI (1 μg/ml), the signal detection and analysis of subsequent metaphases used the Metafer system and Metasytems' ISIS software, respectively. F3-1 and M5-T1 karyotypic formulas were reported using an abbreviated format of the International System for Human Cytogenetic Nomenclature (ISCN), omitting breakpoint information either when the quality of counterstained chromosomes or the blending of colors through fluorescence flaring prevented the accurate definition of breakpoints. Chromosomal gains or structural aberrations had to be detected in at least two metaphases and chromosomal losses in three metaphases to be acknowledged as clonal. Chromosome identification and band nomenclature were indicated according to the standard rat karyotype [73].

### Statistical analyses

Statistical analyses were performed using the Mann-Whitney nonparametric test for comparison of the means, using the Graph-Pad Prism software. A *p* value < 0.05 was considered to be statistically significant. All graphs present the mean and standard deviations of at least three independent experiments.

## SUPPLEMENTARY FIGURES AND TABLES


