# Supplementary material for: Characterization of preneoplastic and neoplastic rat mesothelial cell lines: the involvement of TETs, DNMTs, and 5-hydroxymethylcytosine
Source: Oncotarget. 2016 Apr 25;7(23):34664–87. doi: 10.18632/oncotarget.8970 (PMC5085183; doi:10.18632/oncotarget.8970)
Supplement: Supplementary file 1 [file oncotarget-07-34664-s001.pdf]

SUPPLEMENTARY FIGURES AND TABLES

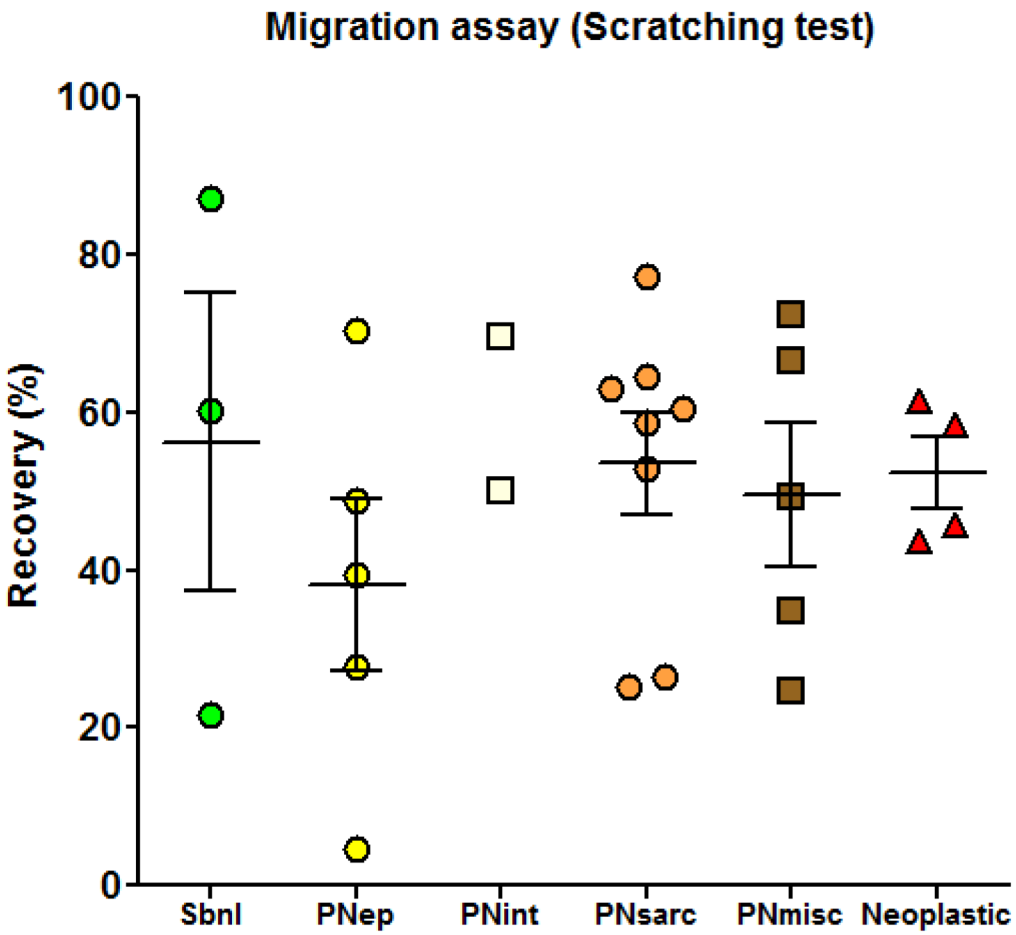

Supplemental Figure S1: Measurement of cell migration. Cell migration was induced by scratching monolayer of cells from the different cell lines of the rat biocollection. When cell density was confluent in 12-well plates (35 mm), wound lines were made by scratching cellular monolayer with a plastic pipette tip. The width of scratch was recorded by taking photographs under phase contrast microscope at 17h, and quantified with ImageJ analysis software. Each dot represent the mean of three experiments for each cell line.

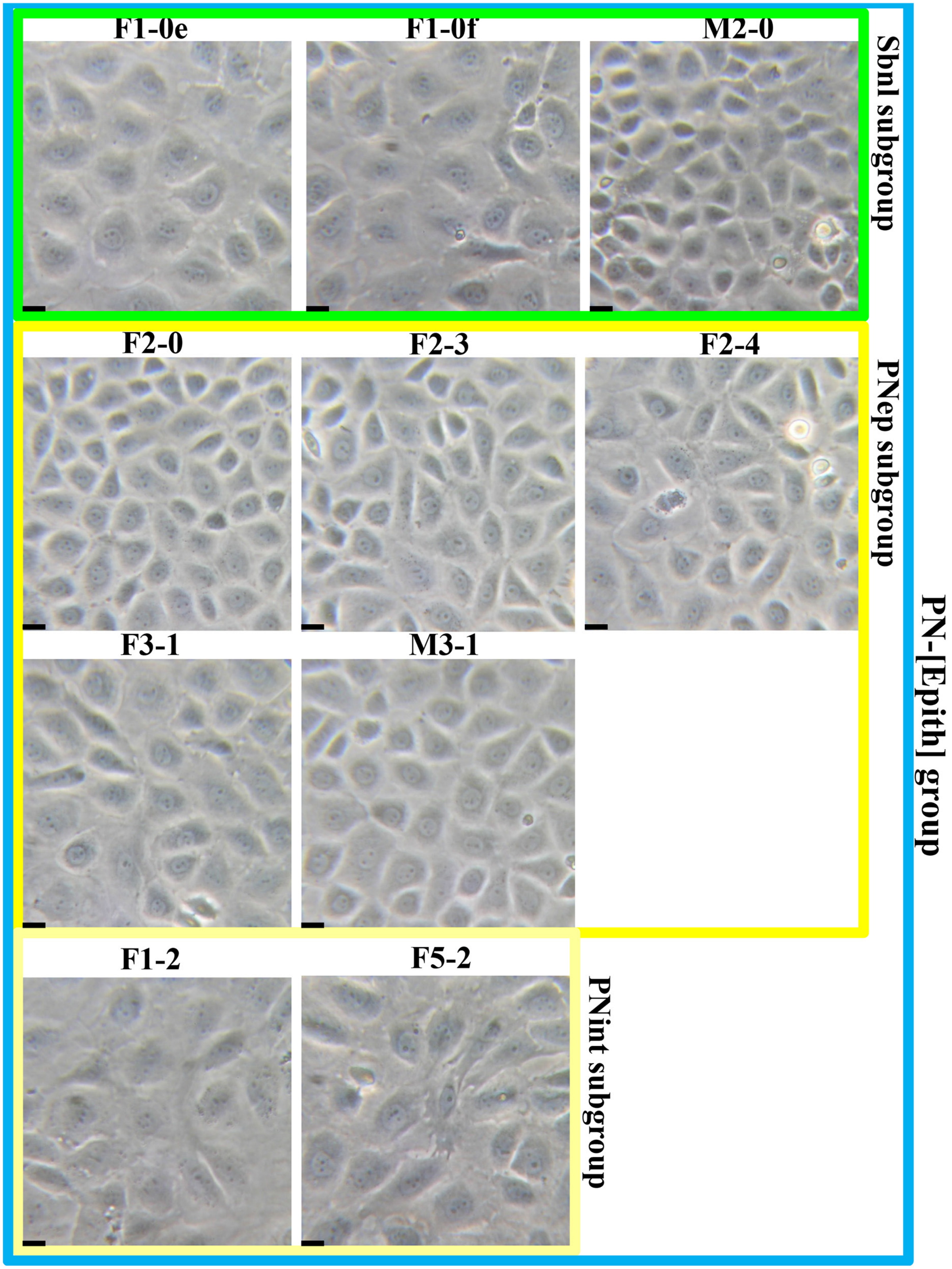
F1-0e
F1-0f
M2-0
Sbnl subgroup
F2-0
F2-3
F2-4
PNep subgroup
F3-1
M3-1
F1-2
F5-2
PNint subgroup
PN-[Epith] group

(*Continued*)

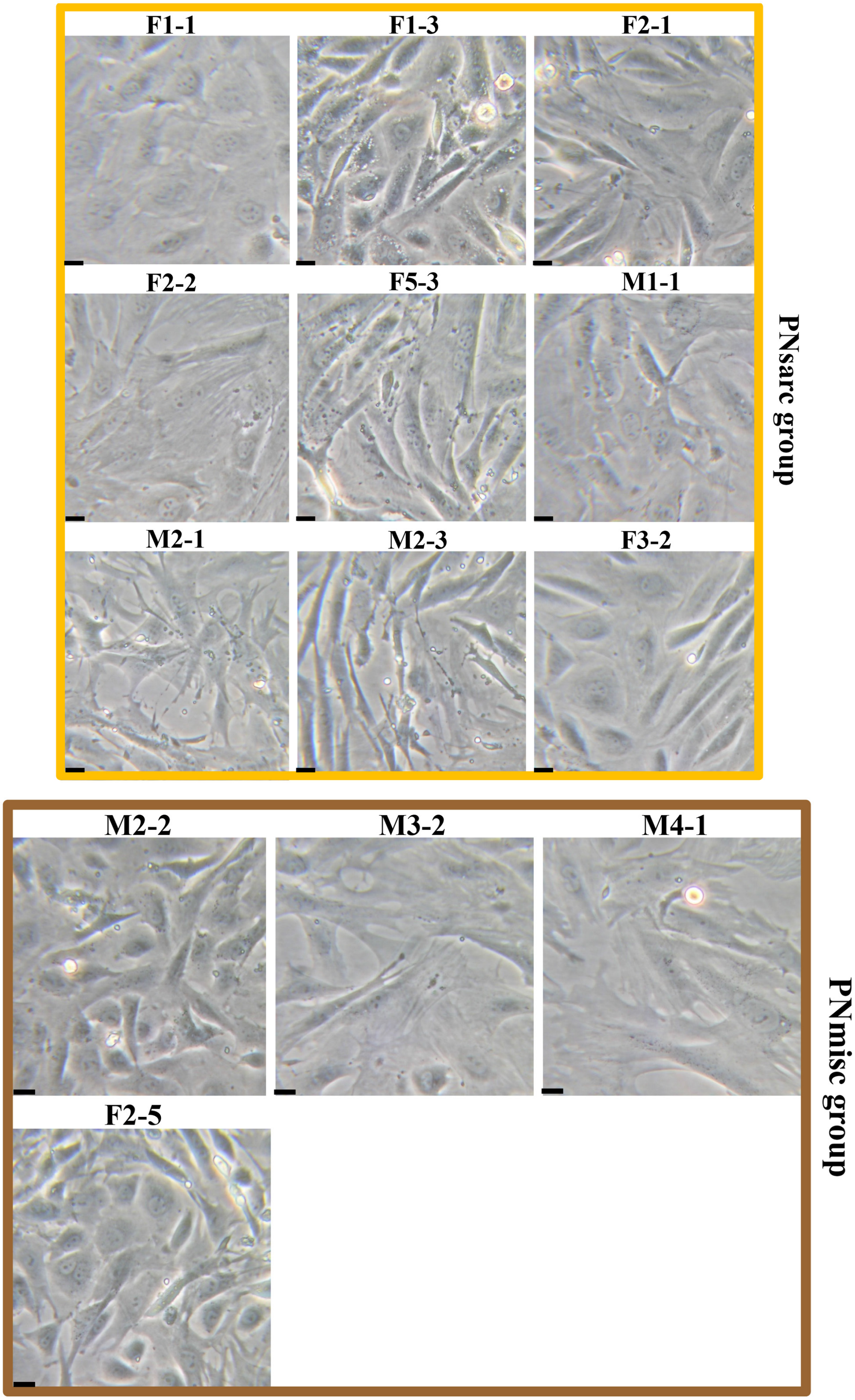
F1-1
F1-3
F2-1
F2-2
F5-3
M1-1
M2-1
M2-3
F3-2
PNsarc group
M2-2
M3-2
M4-1
F2-5
PNmisc group

(*Continued*)

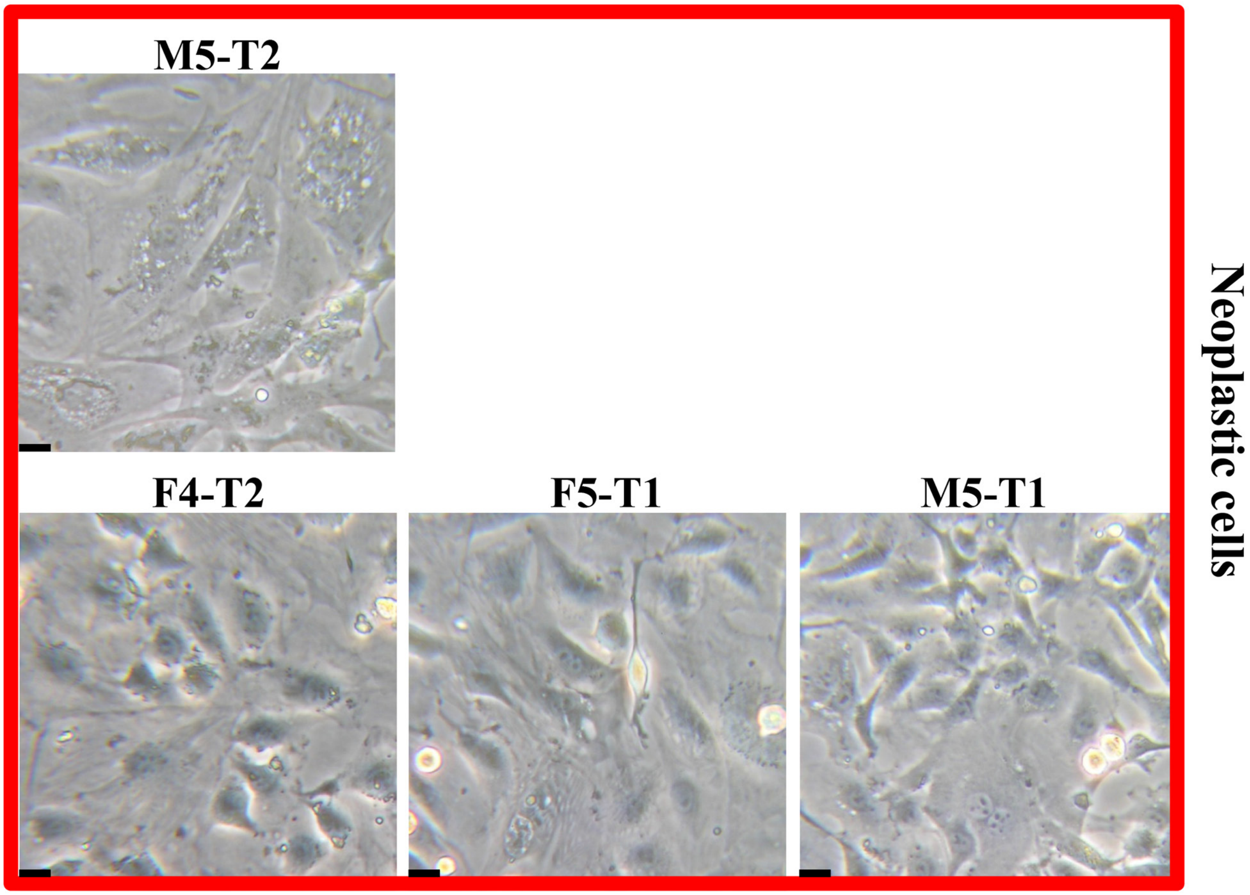

Supplemental Figure S2: Morphology at confluence of the 27 rat cell lines in culture. Morphology of cultured cells for each cell line of the rat biocollection, 6-well plate (96 mm), at confluence. Scale bars represent 10 µm.

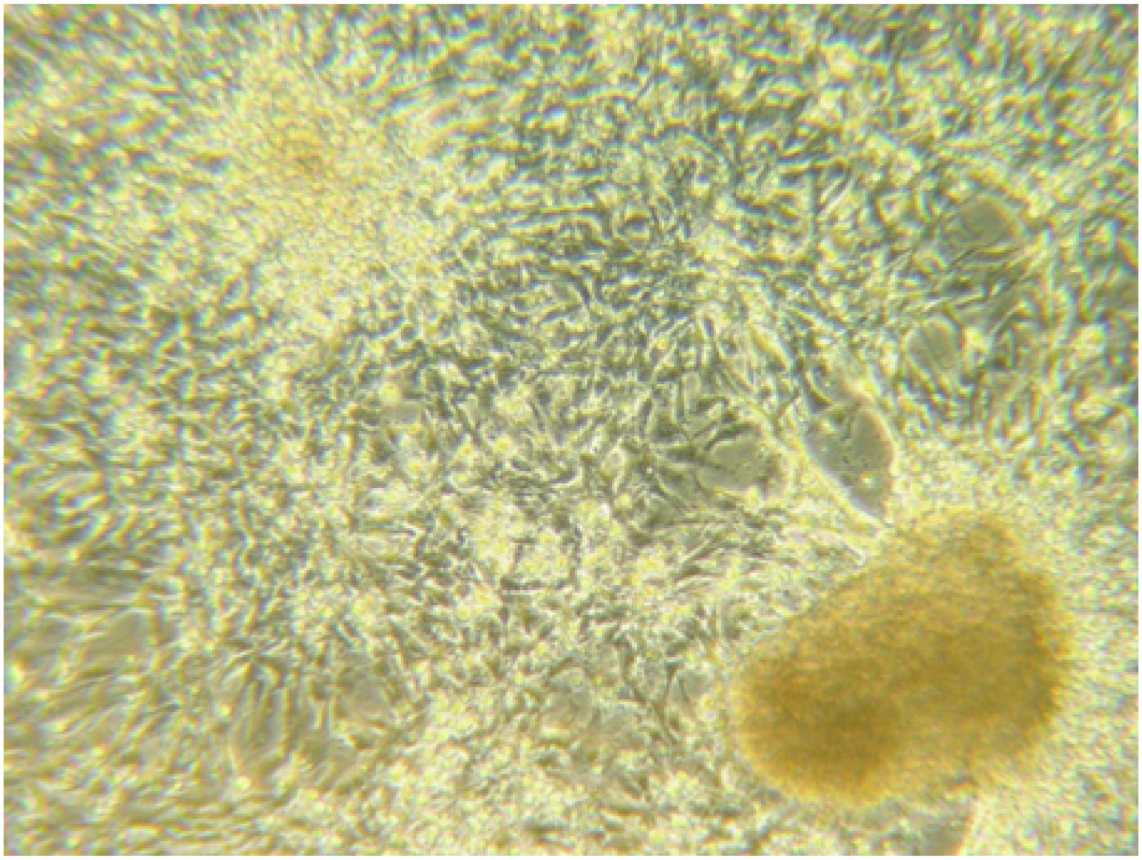

Supplemental Figure S3: Morphology at full confluence of the M5-T1 neoplastic rat cell line with formation of spheroids.

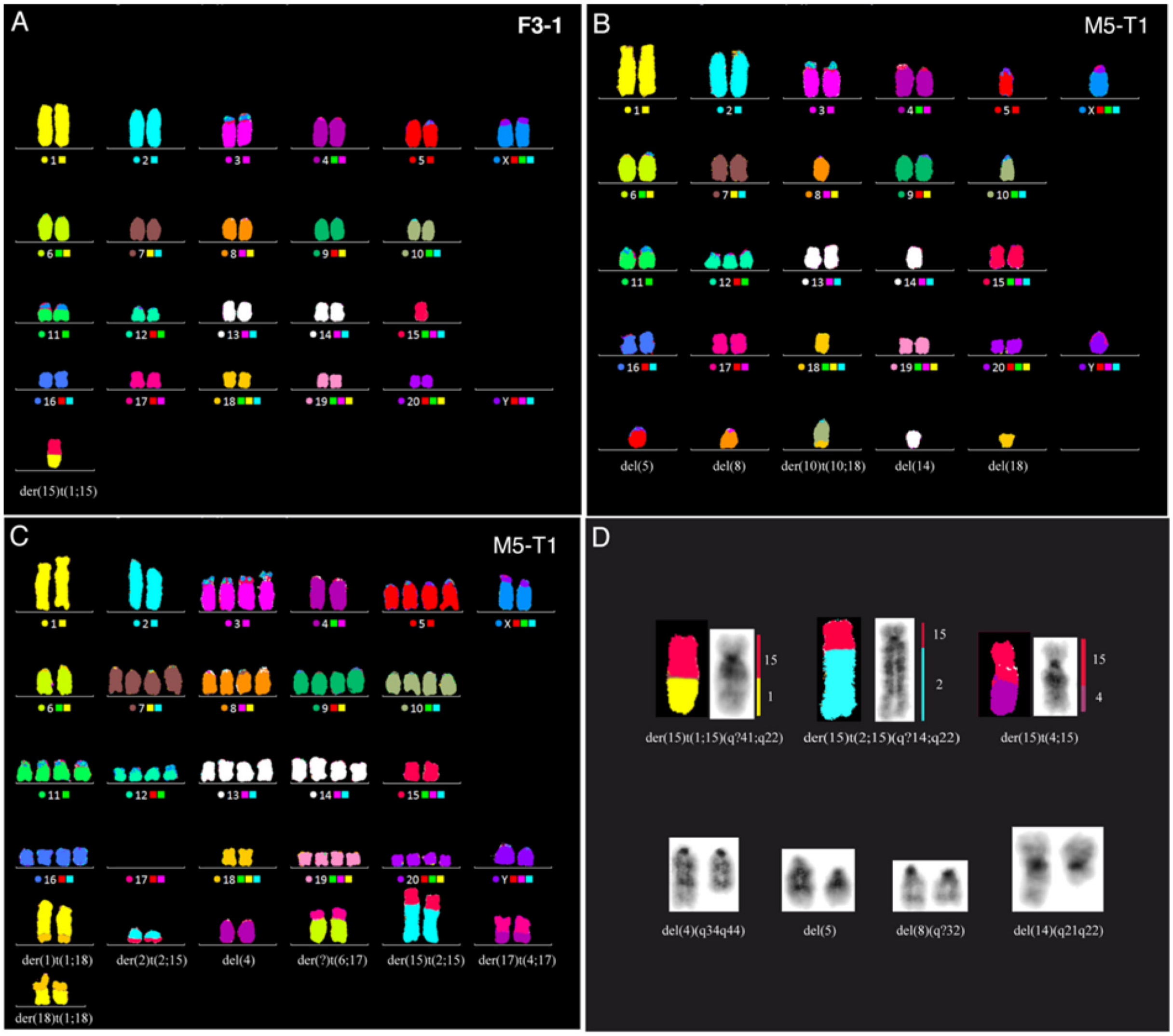

Supplemental Figure S4: Multicolor FISH karyotypes of the F3-1 and M5-T1 cell lines. The F3-1 cell line is representative of the PNep subgroup of epithelioid preneoplastic rat mesothelial cell lines. M5-T1 corresponds to the most aggressive rat mesothelioma cell line.

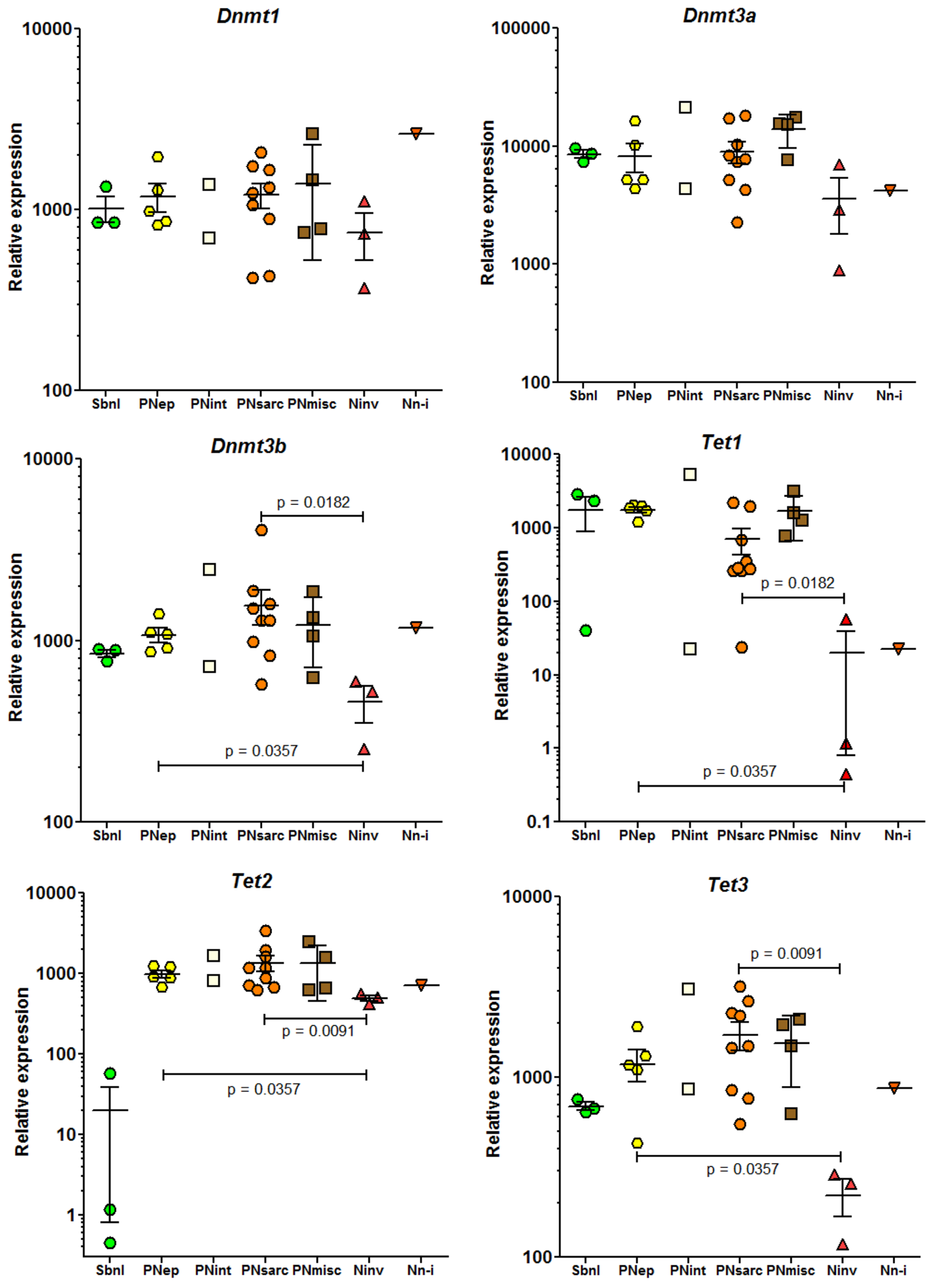

Supplemental Figure S5: RT-PCR analysis of the relative expression of epigenetic enzymes in the different groups and subgroups of preneoplastic rat cell lines. DNA methyltransferase genes *Dnmt1*, *Dnmt3a* and *Dnmt3b*. Ten-Eleven Translocation (TET) dioxygenase genes *Tet1*, *Tet2* and *Tet3*.

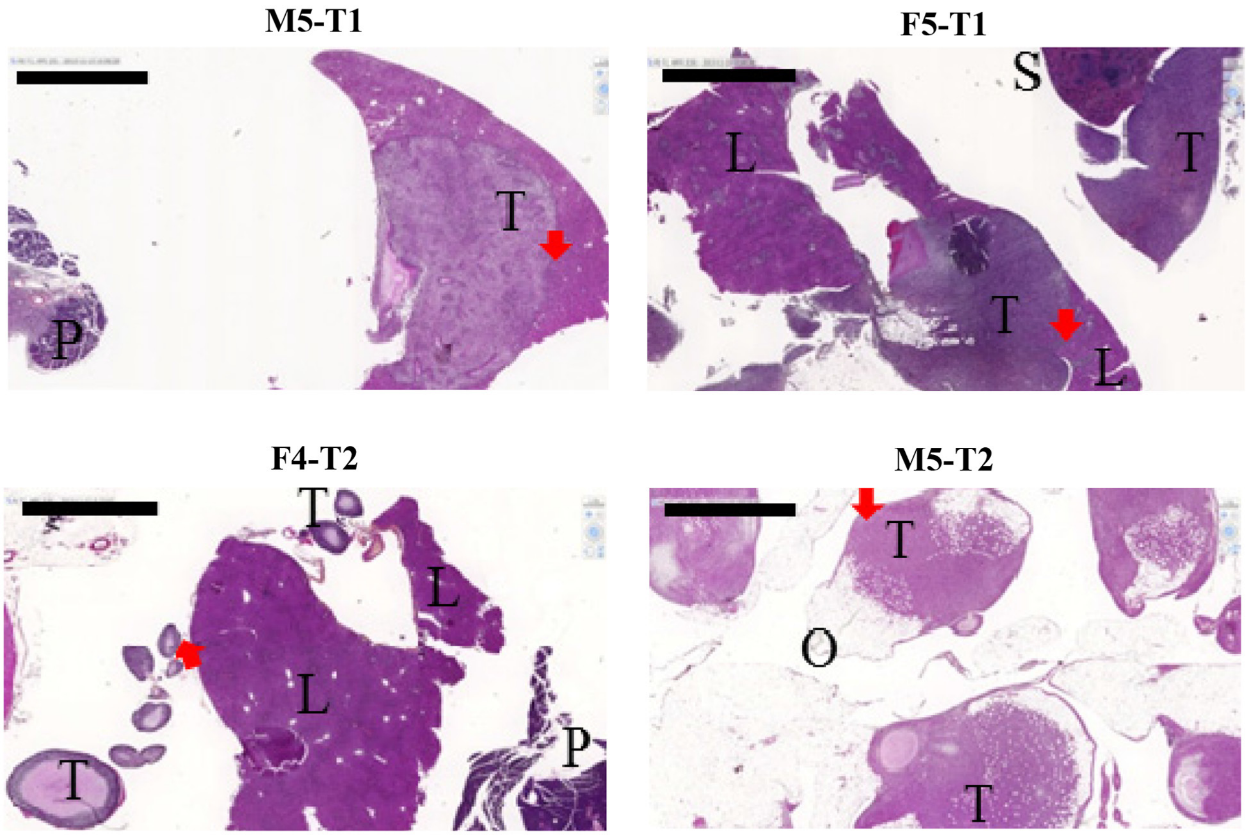

Supplemental Figure S6: General views of the four types of tumor obtained after i.p. transplantation of neoplastic cell lines into syngeneic rats. HPS staining. The scale bars represent 2.5 mm. L, liver, O, omentum. P, pancreas. S, spleen. T, tumor. The red arrows indicate the position of the detailed views of tumor cells given in Figure 8A.

Supplemental Table S1: Characteristics of cell lines in culture (6-well plates, RPMI 1640 medium).

Type	Group	Subgroup	Name	Doubling time (h)	Saturation density ($10^5/cm^2$)
	Epithelioid morphology	Sbnl	F1-0e	45	1.1
			F1-0f	38	1.3
			M2-0	42	3.0
	PN-[Epith]	PNep	F2-0	30	2.0
			F2-3	32	2.6
			F2-4	46	1.3
			F3-1	125	0.9
			M3-1	70	1.4
		PNint	F1-2	90	0.9
			F5-2	41	1.6
Preneoplastic	Sarcomatoid Morphology PNsarc		F1-1	30	1.2
			F1-3	41	1.6
			F2-1	144	0.6
			F2-2	26	1.0
			F5-3	250	1.1
			M1-1	95	0.5
			M2-1	44	2.5
			M2-3	24	0.7
			M3-2	98	0.7
	Miscellaneous PNmisc		M2-2	65	2.0
			M4-1	200	0.5
			F2-5	28	1.0
			F3-2	21	1.2
Neoplastic	Noninvasive		M5-T2	28	1.1
	Invasive		F4-T2	46	2.8
			F5-T1	22	2.3
			M5-T1	18	4.8

Supplemental Table S2: relative expression profiles of the different rat MM cell lines

	Cdkn2a	*Rassf1*	*Wt1*	*Pdpn*	*Ezr*	*Msln*	*Hmgb1*	*Acta 2*	*Zeb1*	*Tgfb1*
**M5-T2**	4.97	1383.57	1751.96	2795.8	815.62	403.14	986.79	1972.27	1713.33	2005.21
**F4-T2**	2.54	653.92	355.00	289.42	529.93	186.93	719.59	31.71	974.46	923.37
**F5-T1**	2.10	442.49	509.78	13.74	224.22	109.62	777.74	29.77	501.71	404.9
**M5-T1**	0.79	628.11	570.37	4.76	751.88	164.28	703.98	0.29	504.07	434.96

Supplemental Table S3: Karyotypic formulas of the F3-1 (PNep subgroup of preneoplastic cell lines) and M5-T1 (neoplastic invasive subgroup) rat experimental cell lines

Cell line		Karyotype
**F3-1**	42, XX, der(15)t(1;15)(q?41;q2?4)[10]/36~43, XX, +12[2], -17[5], +mar[2] cp[18]/42, XX [41]	
**M5-T1**	Diploid metaphases	38~44, XY, ins(1)[4], del(1)[2], der(2)t(1;2)[4], del(4)[5], del(5)[37], del(8)[23], der(10)t(1;10)[5], der(10)t(10;18)[15], +12[37], del(14)[40], der(15)t(1;15)[2], der(15)t(4;15)[6], +17[2], del(18)[13], der(18)t(10;18)[2], -19[3] cp [41]
	Tetraploid metaphases	79~88, XXYY, der(1)t(1;18)x2[7], -2[3], der(2)t(1 ;2)x2[2], der(2)t(2;15)x2[8], del(4)(q34q44)x2[8], del(5)x2[21], der(6)t(6;17)x2[8], del(8)x2[15], 9[3], der(10)t(10 ;18), der(10)t(10;18)x2[4], 11[3], +12[2], +12[20], 13[3], del(14)x2[21], der(15)t(2;15)x2[8], -17, -17[6], der(17)t(4;17)x2[8], del(18)x2[5], der(18)t(1;18)x2[8], -20[5], +mar[4] cp[32]
